# TREAT Early Arthralgia to Reverse or Limit Impending Exacerbation to Rheumatoid arthritis (TREAT EARLIER): a randomized, double-blind, placebo-controlled clinical trial protocol

**DOI:** 10.1186/s13063-020-04731-2

**Published:** 2020-10-16

**Authors:** Ellis Niemantsverdriet, Yousra J. Dakkak, Leonie E. Burgers, Femke Bonte-Mineur, Gerda M. Steup-Beekman, Sjoerd M. van der Kooij, Hido D. Boom, Cornelia F. Allaart, Pascal H. P. de Jong, Annette H. M. van der Helm-van Mil

**Affiliations:** 1 Department of Rheumatology, Leiden University Medical Center, Leiden, The Netherlands; 2 Department of Rheumatology, Maasstad Hospital, Rotterdam, The Netherlands; 3 Department of Rheumatology, Haaglanden Medical Center – Bronovo, The Hague, The Netherlands; 4 Department of Rheumatology, Haga Hospital, The Hague, The Netherlands; 5 Department of Rheumatology, Spaarne Gasthuis, Haarlem, The Netherlands; 6 Department of Rheumatology, Erasmus Medical Center, Rotterdam, The Netherlands

**Keywords:** Rheumatoid arthritis, Clinically suspect arthralgia, Subclinical inflammation, MRI, Intervention, Prevention, Methotrexate, Double-blind, Placebo-controlled, Randomized

## Abstract

**Background:**

We present a study protocol for a randomized, double-blind, placebo-controlled trial that investigates the hypothesis if intervention in the symptomatic phase preceding clinical arthritis (clinically suspect arthralgia (CSA)) is effective in preventing progression from subclinical inflammation to clinically apparent persistent arthritis. Currently, rheumatoid arthritis (RA) can be recognized and diagnosed when arthritis (joint swelling) has become detectable at physical examination. Importantly, at this time, the immune processes have already matured, chronicity is established, and patients require long-standing treatment with disease-modifying anti-rheumatic drugs. The TREAT EARLIER trial studies the hypothesis that intervention in the symptomatic phase preceding clinical arthritis is more often successful in permanent disease modification because of less matured underlying disease processes.

**Methods:**

A two-level definition to identify patients that are prone to develop RA is used. First, patients should have CSA and recent-onset arthralgia (< 1 year) that is suspect to progress to RA according to the expertise of the treating rheumatologist. Second, patients need to have subclinical inflammation of the hand or foot joints at 1.5 T MRI. The trial aims to recruit 230 participants from secondary care hospital settings across the south-west region of The Netherlands. Intervention will be randomly assigned and includes a single-dose of intramuscular 120 mg methylprednisolon followed by methotrexate (increasing dose to 25 mg/week orally) or placebo (both; injection and tablets) over the course of 1 year. Thereafter, participants are followed for another year. The primary endpoint is the development of clinically detectable arthritis, either fulfilling the 2010 criteria for RA or unclassified clinical arthritis of ≥ 2 joints, which persists for at least 2 weeks. DMARD-free status is a co-primary endpoint. The patient-reported outcomes functioning, along with workability and symptoms, are key secondary endpoints. Participants, caregivers (including those assessing the endpoints), and scientific staff are all blinded to the group assignment.

**Discussion:**

This proof-of-concept study is the logical consequence of pre-work on the identification of patients with CSA with MRI-detected subclinical joint inflammation. It will test the hypothesis whether intervention in patients in this early phase with the cornerstone treatment of classified RA (methotrexate) hampers the development of persistent RA and reduce the disease burden of RA.

**Trial registration:**

Dutch Trial Register NL4599 (NTR4853). Registered on 20 October 2014

## Administrative information

**Table:** 

Title {1}	TREAT Early Arthralgia to Reverse or Limit Impending Exacerbation to Rheumatoid arthritis (TREAT EARLIER): a randomized, double-blind, placebo-controlled clinical trial protocol
Trial registration {2a and 2b}.	Dutch Trial Register: NTR4853/Trial NL4599. Registered 20 October 2014, https://www.trialregister.nl/trial/4599.
Protocol version {3}	25-02-2020, protocol version 7
Funding {4}	This trial is financially supported by a ZonMW grant (programma translationeel onderzoek). The remaining costs will be covered by the Department of Rheumatology of the Leiden University Medical Center.
Author details {5a}	E. Niemantsverdriet, study-coordinator, wrote the manuscript and coordinated submission, Department of Rheumatology, Leiden University Medical Center, Leiden, The Netherlands Y.J. Dakkak, study-doctor of the trial, Department of Rheumatology, Leiden University Medical Center, Leiden, The Netherlands L.E. Burgers, study-doctor of the trial, Department of Rheumatology, Leiden University Medical Center, Leiden, The Netherlands F. Bonte-Mineur, rheumatologist who referred eligible patients for this trial, Department of Rheumatology, Maasstad Hospital, Rotterdam, The Netherlands G.M. Steup-Beekman, rheumatologist who referred eligible patients for this trial, Department of Rheumatology, Haaglanden Medical Center - Bronovo, The Hague, The Netherlands S.M. van der Kooij, rheumatologist who referred eligible patients for this trial, Department of Rheumatology, Haga Hospital, The Hague, The Netherlands H.D. Boom, rheumatologist who referred eligible patients for this trial, Department of Rheumatology, Spaarne Gasthuis, Haarlem, The Netherlands C.F. Allaart, trial-advisor and revised the manuscript, Department of Rheumatology, Leiden University Medical Center, Leiden, The Netherlands P.H.P. de Jong, trial-advisor and revised the manuscript, Department of Rheumatology, Erasmus Medical Center, Rotterdam, The Netherlands A.H.M. van der Helm-van Mil, designed and supervised the study, contributed to writing of the manuscript and revised the manuscript, Department of Rheumatology, Leiden University Medical Center, Leiden, The Netherlands, and Department of Rheumatology, Erasmus Medical Center, Rotterdam, The Netherlands
Name and contact information for the trial sponsor {5b}	Investigator-initiated clinical trial Principal investigator: Annette H.M. van der Helm-van Mil Leiden University Medical Center, Albinusdreef 2, 2333 ZA Leiden, The Netherlands.
Role of sponsor {5c}	Principal investigator contributed in study design, collection, management, and will contribute to analysis, interpretation of data, writing the report, and the decision to submit the report for publication. The funding bodies did not have any role in study design, collection, and management, nor will have any role in analysis, interpretation of data, writing the report, or decision to submit the report for publication.

For more detailed information we refer to the WHO trial registration data set: supplementary file 1.

## Introduction

### Background and rationale {6a}

Rheumatic and musculoskeletal diseases (RMDs) are among the most prevalent, disabling, and burdensome non-communicable diseases in Europe and the USA, eliciting high costs for healthcare and social security budgets. RMDs are also the number one cause of disability in Europe. Rheumatoid arthritis (RA) is the commonest inflammatory cause of disability. It affects 1% of the population and is characterized by inflammation and destruction of the joints; inflammation is typically located in the hand and foot joints, but other joints are also regularly inflamed. Pain, stiffness, activity limitation, and functional disability are direct consequences of inflammation and have an impact on physical functioning on the individual level and result in work loss. These disease aspects are the worst at the time of RA diagnosis and do not revert to normality when treatment is initiated during the phase of clinical arthritis [1–3]. This causes a large burden of costs for society; direct costs have considerably escalated due to expensive therapies, and indirect costs are due to sick leave, work loss, and disability pensions [4]. New biologic drugs and better treatment strategies have resulted in improved disease outcomes during the last decades, and clinically relevant joint destruction has become infrequent. However, RA patients still have a chronic disease and RA remains a remitting and relapsing condition that cannot be cured. The ultimate treatment strategy prevents the development of persistent RA.

The mechanisms underlying arthritis becoming chronic are poorly understood. The development of RA is considered to be a multiple-hit process that is largely taking place before the disease presents with clinically swollen joints. Different phases in the development of RA are described by a “European League Against Rheumatism” (EULAR) taskforce [5, 6]; the phase that precedes the development of clinically apparent arthritis is a phase of symptoms. The pattern of symptoms that characterizes this pre-arthritis phase has been called clinically suspect arthralgia (CSA) [7]. The clinical expertise (relying on pattern recognition) of rheumatologists has been shown accurately in the identification of patients with arthralgia at risk for RA among all patients with arthralgia presenting to secondary care (odds ratio 55, sensitivity 80%, specificity 93%) [8]. In this phase of CSA, patients have not only symptoms such as pain and stiffness but also functional limitations [9]. Sick leave and disability pensions are described to rise 6 months before the diagnosis [4]. Additionally, patients can have maturing auto-antibody response or increased levels of pro-inflammatory cytokines in the systemic circulation [10, 11]. Together, these findings illustrate the relevance of the CSA phase from the patients’ perspective and the immunological perspective.

Although clinical joint swelling is per definition absent in CSA, patients can have subclinical joint inflammation in the hands or feet, that is, synovitis, tenosynovitis, and/or inflammation of the subarticular bone, also called osteitis or bone marrow edema (BME). Magnetic resonance imaging (MRI) is a sensitive tool to detect subclinical joint inflammation. Furthermore, the definition of the presence of (“positivity for”) subclinical joint inflammation was established by the generation of a reference atlas from persons from the general population [12, 13]. Including information on MRI findings from age-matched symptom-free persons (same feature, same anatomic location), a highly specific definition was obtained without losing sensitivity. The presence of subclinical inflammation in patients with CSA is associated with a risk of 32% to progress to RA during the next year [14]; the NPV is 93%, indicating an acceptable ability to detect RA in a symptomatic pre-arthritis phase and a low chance to miss patients because of false-negative results.

The risk of progression to clinical arthritis and RA is an ongoing research area. At present, no internationally validated prediction model is available. Patients with CSA have a higher risk of RA development than patients with non-specified musculoskeletal symptoms [15]. In addition to subclinical inflammation, the presence of auto-antibodies (anti-citrullinated peptide antibody (ACPA) and rheumatoid factor (RF)) is associated with the development of clinical arthritis [16]. The data on the predictive value of ACPA levels are contradictory [16]. Also, the predictive value of inflammatory response proteins measured in the systemic circulation is unclear; most research has so far been conducted on C-reactive protein (CRP), which is routinely measured in the clinic, but its predictive value in arthralgia is not undisputed [16]. Thus, the presence of imaging-detected subclinical inflammation and auto-antibodies are two validated and independent risk factors in CSA. Importantly, MRI-detected subclinical inflammation yields elevated PPVs for RA development also in ACPA-negative CSA patients [17, 18].

There are five other placebo-controlled trials aiming at secondary prevention, namely PRAIRI (2009-010955-29), APIPPRA (2013-003413-18), ARIAA (2014000555-93), and STAPRA (2013-05524-42) reported on www.clinicaltrialsregister.eu, and StopRA (NCT02603146) reported on www.ClinicalTrials.gov. STAPRA has been stopped prematurely, which studied the effect of intervention with statins. The other ongoing trials study the effectiveness of intervention with biologic disease-modifying anti-rheumatic drugs (DMARDs). All mentioned trials include participants positive for auto-antibodies. The TREAT EARLIER trial will include patients with CSA irrespective of the auto-antibody status. While ACPA-positive has always been a more severe subset of RA, up-to-date treatment strategies have made the disease burden comparable in many aspects for ACPA-positive and ACPA-negative patients with RA [19]. This implies that efforts to further improve the disease outcomes should be proportional to both disease subsets. Furthermore, for the management of early arthritis, EULAR recommends methotrexate (MTX) as the first-line therapy, possibly combined with a short course of corticosteroids, independent of the auto-antibody status [20]. Based on observations done in patients with classified RA (in both APCA-positive and ACPA-negative RA) that the time to intervention determines the efficacy of intervention [21] and the fact that subclinical joint inflammation can now be detected in patients with CSA, we hypothesize that intervention with the cornerstone and first-line therapy of RA in this pre-arthritis phase is effective in preventing arthritis becoming chronic (“chronification”). The present proof-of-concept study will test this hypothesis with our ultimate goal to reduce the disease burden of RA.

### Objectives {7}

**Fig. 1:**
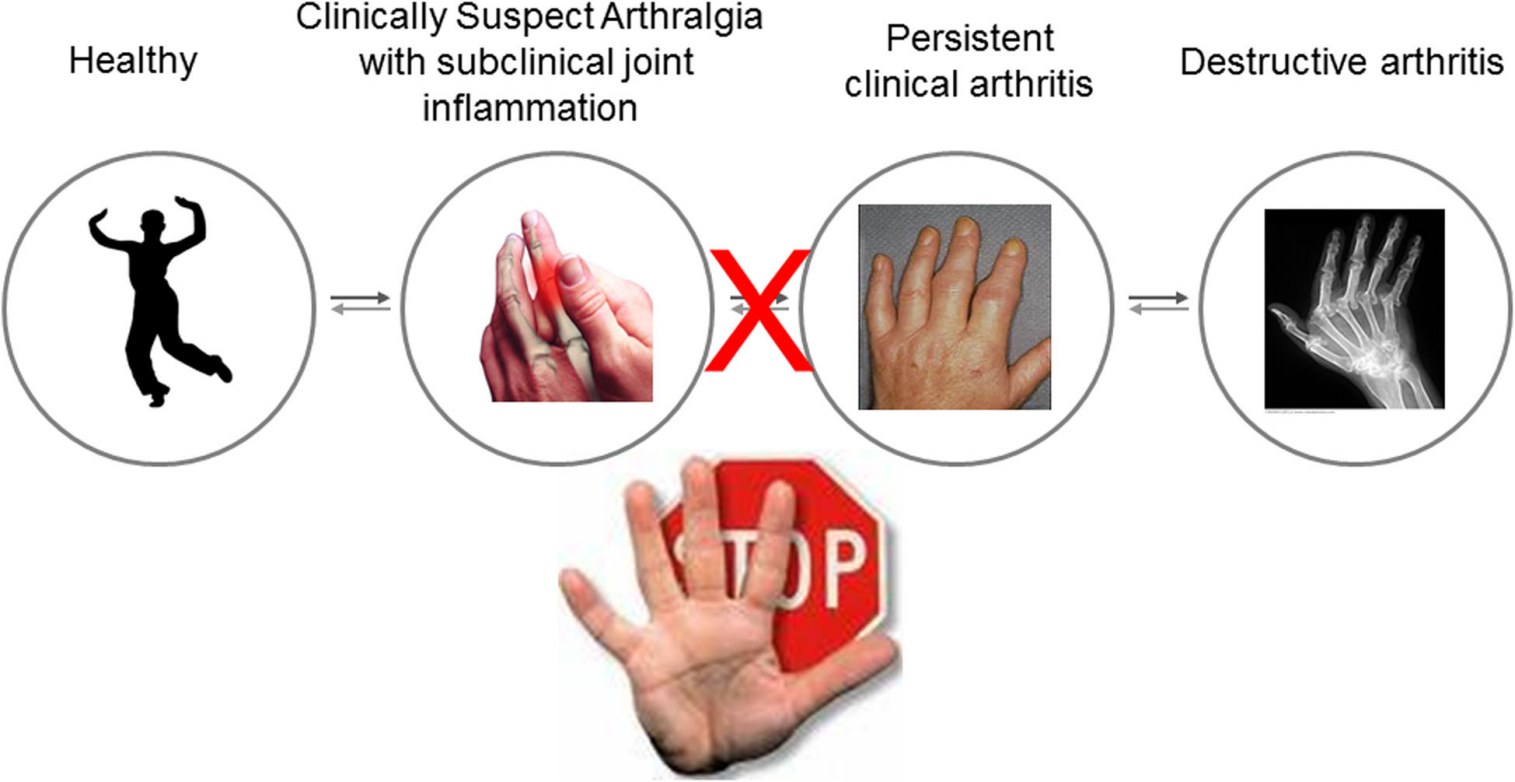
Schematic figure of the objective of the TREAT EARLIER trial

We hypothesize that intervention in the pre-arthritis phase of CSA with subclinical joint inflammation is effective in preventing arthritis from becoming chronic (“chronification”). The present proof-of-concept study will test this hypothesis with our ultimate goal to reduce the disease burden of RA (Fig. 1).

### Trial design {8}

**Fig. 2:**
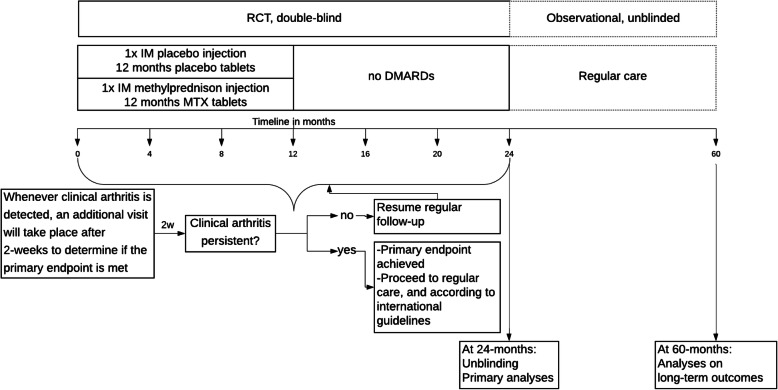
Overview of the TREAT EARLIER study

The present study concerns a randomized placebo-controlled, parallel, double-blind, superiority clinical trial (RCT) comparing an 1-year course of MTX combined with a single glucocorticoid intramuscular (IM) depot injection, with a combination of oral and IM placebo in patients who are deemed to be at high risk of developing RA because of having recent-onset CSA and MRI-detected subclinical inflammation (Fig. 2). Participants will be treated for 1 year and followed for another year to observe the development of clinically detectable arthritis, either fulfilling the 2010 criteria for RA [22] or unclassified clinical arthritis with a 66-swollen joint count (SJC) of ≥ 2, which persist for at least 2 weeks. Participants who reach the primary endpoint will be treated according to the general rheumatologic care and in line with the Dutch and international guidelines [23].

## Methods: study setting, participants, and criteria

### Study setting {9}

The Leiden University Medical Center (LUMC, The Netherlands) is the only center where participants will be enrolled in this single-center study. Screening with MRI will be done in the LUMC, and for the duration of the trial, all participants will be treated in the LUMC. This is done for practical reasons; it allows consistency in the use of MRI (same protocol, same scanner, same readers) in a patient-friendly manner because of excellent accessibility to MRI in the LUMC. It also allows consistency in the determination of endpoints because all data are collected by a group of rheumatologists and research nurses that were trained together.

**Fig. 3:**
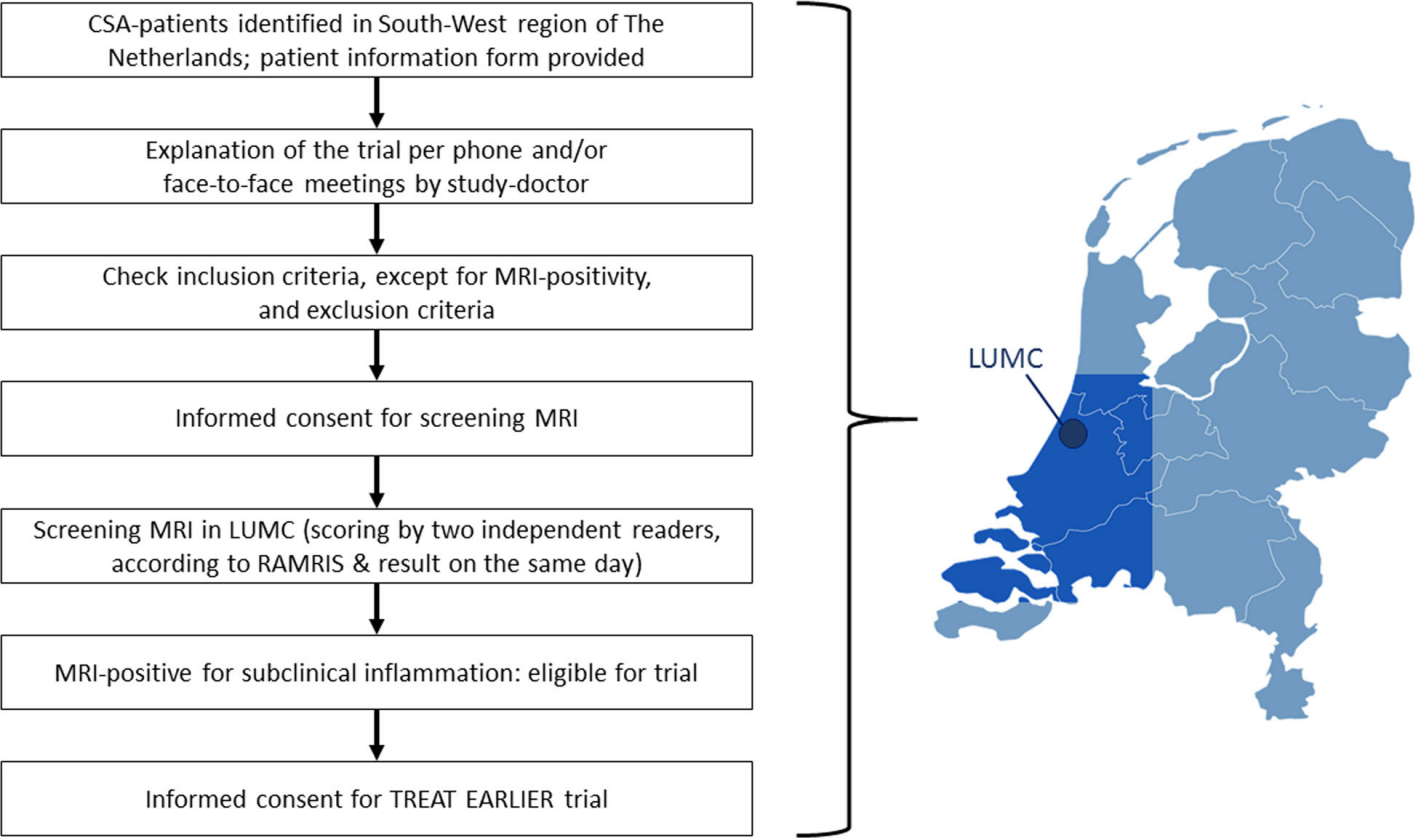
Screening of eligible patients in the southwest region of The Netherlands

Despite the fact that the trial will be performed in one center, recruitment will be done in the South-West region of The Netherlands. Rheumatologist in all hospitals in this region (for a list, see supplementary file 2) have agreed to inform all eligible patients with CSA on the TREAT EARLIER trial and, with the consent of the patient, to transfer the patient’s contact details to the study doctor in the LUMC (Fig. 3). The study doctor, who should have obtained the medical doctor degree (MD) and is working as a PhD student at The Department of Rheumatology within the LUMC, is then responsible for the screening process. The participation of the total region is an important success factor to identify enough patients in this very early phase, because patients presenting with CSA are relatively infrequent as the majority of patients already present with swollen joints. Importantly, our long-standing efforts to allow very early access for patients with CSA [24] and the commitment of all hospitals in the region allows to screen and include sufficient participants in this trial.

### Eligibility criteria {10}

We use a two-level definition to identify patients that are prone to develop RA. First, patients need to have arthralgia that is suspect to progress to RA according to the treating rheumatologist, and second, patients should have a 1.5-T MRI showing local subclinical inflammation (Fig. 3).

#### Clinically suspect arthralgia

No single symptom is highly characteristic and specific for a preclinical phase of RA. However, patients with symptoms at risk for the development of RA, in the absence of clinical arthritis, can be identified by rheumatologists using pattern recognition. This symptom complex is also called CSA [25]. For inclusion in our trial, participants need to have CSA, which is arthralgia of the small joints and of recent onset (symptoms < 1 year) that according to the judgment of experienced rheumatologists are suspect to progress towards RA over time. The presence of symptoms or signs that make other diagnoses more likely rules out CSA. We previously demonstrated that only 6% of all patients that presented with arthralgia to the rheumatology outpatient clinic of the LUMC had CSA, and the clinical expertise had an odds ratio of 55 for identification of patients at risk for RA [8].

The recognition of the symptom complex that is described as CSA followed the identification of different disease phases that precede the development of RA by the EULAR taskforce in 2012 [5]. According to this model, there are genetic and environmental risk factors that play a role where patients are initially asymptomatic; thereafter, symptoms occur and eventually, clinical arthritis develops. After the recognition of CSA, a subsequent EULAR taskforce has derived a definition of arthralgia suspicious for the progression of RA. This definition was derived in 2016 and validated thereafter [7, 26]. The TREAT EARLIER trial was designed in 2014, and therefore, patients with CSA need to be identified by rheumatologists using their pattern recognition, but the fulfillment of the EULAR definition is not a requirement as this definition did not exist at the study start.

Hence, CSA is identified based on clinical symptoms and signs at the first presentation to the outpatient clinic. The results of laboratory investigations are not required for the identification of CSA. Moreover, laboratory assessments are generally not performed by our general practitioners, as according to the Dutch guidelines for primary care general practitioners are discouraged to perform these tests (https://www.nhg.org/standaarden/volledig/nhg-standaard-artritis).

#### MRI-detected subclinical inflammation in small joints

Secondly, CSA patients need to have an MRI positive for subclinical inflammation, which is inflammation that is present in < 5% of the age-matched general population, according to two independent readers blinded to clinical data.

#### MRI protocol

The screening MRIs will be made of the unilateral wrist, metacarpophalangeal (MCP)2–5 joints, and metatarsophalangeal (MTP)1–5-joints at the most painful side, or the dominant side in case of equally severe symptoms. MRIs will be performed on a musculoskeletal (MSK) 1.5-T MRI system (GE, WI, USA) using a 145-mm coil for the foot and a 100-mm coil for the hand at the Department of Radiology of the LUMC. The participants are positioned in a chair beside the scanner, with the hand or foot fixed in the coil with cushions. For a detailed scan protocol, refer to supplementary file 3.

The MRIs will be scored for synovitis, tenosynovitis, and BME (osteitis). Synovitis and osteitis are scored according to the “outcome measures in rheumatology clinical trials” (OMERACT)-RA MRI scoring (RAMRIS) system [27], which is adopted to also include the MTPs. Tenosynovitis in the MCP, MTP joints, and the wrist, with post-contrast sequences, is scored as described by Haavaardsholm et al. [28]. Contrast enhancement around the extensor tendon sheaths in the MCPs are scored according to this method [29, 30]. For a detailed overview, refer to supplementary file 4.

#### MRI reading

To have two trained MRI readers that are available all days to drop their work immediately when an MRI is made to prioritize MRI scoring, every day a week during the expected inclusion duration of 5 years, we have trained 10 MRI readers. All readers have become experienced; they have undergone a training period of 6 months of almost daily MRI reading and have scored hundreds of MRIs from CSA and early arthritis patients as well as healthy controls before finishing the training period. The interreader and intrareader intraclass correlation coefficients (ICCs) are all > 0.90 (supplementary file 5). Furthermore, during the screening period, they will repeatedly determine their intrareader ICCs.

##### Inclusion criteria

1. Age ≥ 18 years.
2. Patients without clinically detectable arthritis but with arthralgia of the small hand or feet joints of recent onset (< 1 year) that according to the rheumatologist is suspect to be an early presentation of RA (this symptom complex is called CSA).
3. Unilateral MRI of the hand and foot joints, positive for subclinical inflammation.
4. Ability and willingness to give written informed.

##### Exclusion criteria

1. Symptoms or signs making diagnoses other than RA more likely. These are among others; > 6 tender points or Heberden or Bouchard nodules (the presence of such characteristics precludes CSA).
2. Presence of, or history of, clinically apparent arthritis (this precludes CSA).
3. Previous or current treatment with DMARDs or corticosteroids (this precludes CSA).
4. Contraindications for MRI: certain metal implants, pacemakers, GFR < 30 ml/min.
5. Pregnancy or the wish to become pregnant, breastfeeding.
6. Bone marrow hypoplasia.
7. Elevated hepatic enzyme levels (ASAT, ALAT > 3 times normal value).
8. Serum creatinine level > 150umol/l or estimated clearance of < 60%.
9. Serious infections such as hepatitis, pyelonephritis in the past 3 months, or chronic infectious diseases such as chronic chest infections with bronchiectasis.

### Who will take informed consent? {26a} and additional consent provisions for collection and use of participant data and biological specimens {26b}

Informed consent will be obtained by research nurses, who are Good Clinical Practice (GCP)-certificated. Informed written consent for screening MRIs will be obtained from all eligible CSA patients (Fig. 3). In case the MRI scan is positive and patients are willing to participate in the trial, the trial informed consent will be signed.

## Methods: interventions

### Explanation for the choice of comparators {6b}

There is no biological data on the mechanisms involved in the “chronification” of arthritis, and scientific evidence to prioritize one specific DMARD is therefore lacking. The EULAR recommendation for the management of RA suggests the use of MTX with or without glucocorticoids as the first choice induction therapy for patients with newly diagnosed RA [23]. MTX in a dosage of ≥ 20 mg/week with folate substitution has become the anchor drug for RA because of its efficacy [20, 31–34], acceptable toxicity profile, and higher retention rate than other DMARDs. MTX is the anchor drug for RA; it has a favorable benefit/risk ratio and is cheap; we propose to study the effectiveness of MTX in patients with CSA. EULAR guidelines for the treatment of RA are similar for auto-antibody-positive and auto-antibody-negative RA patients [20]. Likewise, intervention in the TREAT EARLIER trial will not be stratified for auto-antibody status.

Whether IM corticosteroids are disease-modifying drugs is a matter of debate. They can reduce radiological progression; whether they are able to modify the persistency of the disease is not clear. The SAVE trial showed no effect of a single glucocorticoid injection on the disease course [35], whereas the STIVEA trial reported that a 3-week course of IM glucocorticoid injections resulted in a significantly higher rate of DMARD-free sustained remission compared to placebo injections [36]. These data may indicate that the effect of one or a few IM corticosteroid injections, without subsequent DMARD therapy, on arthritis persistence is not large. In clinical trials such as the FINRA-Co study and the BeSt study, however, it has been demonstrated that combination therapy of MTX with oral prednisone is superior in early symptom suppression and prevention of radiological damage compared to MTX monotherapy. The tREACH trial showed that in undifferentiated arthritis, the initial treatment with a combination of MTX with corticosteroids was also superior to MTX monotherapy, without a difference between oral prednisone or an IM depot injection [37]. The additive effect of low-dose corticosteroids to conventional DMARDs was also described in a recent systematic literature review [38]. Based on these observations, EULAR guidelines recommended that corticosteroids can be added at the start, especially as MTX is slow acting and a short course of corticosteroids can have a rapid effect. Moreover, it has been shown that this addition does not affect safety [39]. Thus, in line with previous and current EULAR recommendations of the management of RA [20, 23, 33], in the current trial, corticosteroids will be added to MTX in the form of a single IM depot injection at the start of MTX.

### Intervention description {11a}

In this double-blind RCT, participants will be randomized to two arms in an 1:1 ratio. The randomization consists of one IM glucocorticoid injection (120 mg methylprednisolone or IM placebo injection) followed by 12 months of MTX tablets (or placebo tablets) (Fig. 2). No other corticosteroid interventions will be allowed during the trial, unless participants have reached the primary endpoint and proceeded to open-label DMARD therapy.

MTX (or placebo) tablets will be increased in 4 weeks to 10 tablets/week (25 mg/week). Open-label folic acid supplementation (5 mg/week) will be added, in line with the Dutch guidelines of MTX use (https://www.nvr.nl/richtlijnen/nvr-richtlijnen-standpunten-en-zorgpaden/). After 45 weeks of 10 tablets/week (MTX or placebo), tablets will be tapered for 4 weeks and then stopped at week 53. Participants will be followed for another 12 months.

### Criteria for discontinuing or modifying allocated interventions {11b}

Laboratory tests to monitor possible side effects of MTX will be performed according to the Dutch guidelines of MTX (https://www.nvr.nl/richtlijnen/nvr-richtlijnen-standpunten-en-zorgpaden/). In case of mild side effects of MTX, either symptom experienced by the participants or abnormalities in the laboratory tests (mild increase in ASAT, ALAT, or mild decrease of leucocyte or thrombocyte counts), the dosage will be decreased to 5 mg (2 tablets/week), until an acceptable status is obtained. In case the dosage will be less than 3 tablets/week (< 7.5 mg MTX/week), MTX is stopped as in daily practice dosages lower than 7.5 mg/week is considered not effective. Then, participants will be treated according to the treating rheumatologist opinion, however not with DMARDs or corticosteroids as this would be a protocol violation. In case of severe side effects, the treating rheumatologist will stop the MTX (or placebo). Also, this is in line with the Dutch guidelines and with the common practice of MTX treatment in RA. The intention of the trial is to follow all participants for the total trial period of 24 months. Participants who need to stop the study medications because of toxicity (or other reasons, e.g., wish of participant) will be asked to remain in the trial for the entire follow-up period.

### Strategies to improve adherence to interventions {11c}

MTX is considered as a chemotherapy medication (despite the low dose of the tablets of 2.5 mg each), and therefore, it is not allowed for researches to count tablets at the moment of tablet distribution and return, according to our pharmacy guidelines. Therefore, participants will be asked to complete a medication diary in order to monitor medication adherence, which will be done for study medication and concomitant non-steroidal anti-inflammatory drugs (NSAIDs) use (and will be reviewed every visit).

### Relevant concomitant care permitted or prohibited during the trial {11d}

#### Concomitant and prohibited treatment

Concomitant treatment with analgesics such as acetaminophen and/or NSAIDs will be allowed for all participants, except 24 h before MRI scans [40]. Treatment with DMARDs (conventional synthetic, biologic DMARDs, or jak-stat inhibitors) or corticosteroids (systemic or intra-articular) will be prohibited during the trial, unless participants reached the primary endpoint of the trial. Also, any other medicinal products that, in the supervising physician’s opinion, may influence underlying disease activity through effects on immune or inflammatory responses, or both, are prohibited (with the exception of NSAIDs and acetaminophen).

All participants will be evaluated for safety. Screening on toxicity on MTX will be performed by the treating rheumatologists in line with the Dutch guidelines on MTX as recommended by the Dutch Society for Rheumatology (https://www.nvr.nl/richtlijnen/nvr-richtlijnen-standpunten-en-zorgpaden/).

#### Concomitant care

Study visits will be scheduled every 4 months for 2 years follow-up; however, in case a participant has an increase in symptoms in between two study visits, he/she can be seen for an additional visit by their rheumatologist (Fig. 2). In case clinically apparent inflammatory arthritis is detected, an additional study visit will take place after 2 weeks (and in between the already scheduled study visits), to check if a participant has reached the primary endpoint, which is solely based on physical joint examination. Except for MRI positivity/negativity during screening, MRI results during the trial will not be communicated to participants and rheumatologists. Ultrasound will not be made. Hence, evaluation of the primary endpoint is not influenced by the results from imaging.

### Provisions for post-trial care {30}

Participants who reach the primary endpoint may proceed to open-label DMARD therapy, which will be prescribed by their rheumatologist, and followed through routine clinical practice. After these first 2 years, the RCT will be stopped (and unblinded) and participants will be followed for another 3 years under observational extension based on regular care (Fig. 2). During this period, participants who have not achieved the primary endpoint at month 24 can remain under rheumatologic follow-up. This will be left to the decision of the treating rheumatologists and participant (shared decision-making healthcare). If routine follow-up at the rheumatology outpatient clinic is then stopped, participants will be provided with the contact details of the research team to ensure prompt reporting of new signs and symptoms of inflammatory arthritis. In addition, at month 60, all participants will be contacted again.

All patients are insured during the trial or when an injury (as a consequence of the trial) originates within 4 years after the trial.

### Methods: outcomes {12}

The key primary endpoint is the development of clinically detectable arthritis, either fulfilling the 2010 criteria for RA [22] or unclassified clinical arthritis with SJC-66 of ≥ 2 joints, which persists for at least 2 weeks. This primary endpoint (incidence) will be obtained during a 2-year follow-up.

The presence of clinical arthritis (swollen joints) is based on the physical evaluation of the joints by rheumatologists. In case of doubt on the presence of arthritis, it will not be scored. At the start of the trial, all treating rheumatologists attended a session to verify the comparability of determining the endpoint. They demonstrated to have a high interindividual agreement in the evaluation of the presence or absence of clinical arthritis at the patient level. The primary endpoint is not influenced by results from imaging. Ultrasound will not be done. MRIs made during the trial will be stored under a randomization number, and the results will not be reported back to participants or rheumatologists. Thus, also the MRI results are not included in the decision making on the clinical endpoint.

The co-primary endpoint is DMARD-free status. This is assessed at the 2-year follow-up time point and is defined as the absence of clinically detectable synovitis at the joint examination in the absence of DMARD use (including systemic or intra-articular glucocorticoids).

A key secondary endpoint is functioning (Health Assessment Questionnaire Disability Index (HAQ-DI) range 0–3); this will be assessed together with other patient-reported outcomes (PROs) that are interrelated but for which no composite measure exists:

- Work productivity and impairment scale (WPAI) (per item percentage/number and numeric rating scale (NRS) range 0–10).
- Symptoms: pain (NRS range 0–10), fatigue (NRS range 0–10), and morning stiffness (NRS range 0–10, duration in minutes).

Explorative endpoints:

- Tender joint count (TJC-68, range 0–68).
- Hand function: ability to make a fist (left and right, yes/no), squeeze test (hand, foot, left and right, positive/negative), and grip strength test with a dynamometer (range 0–90).
- Global assessment (NRS range 0–10).
- Quality of life: short form-36 (SF-36) (range 0–100), EuroQol 5D-5level (EQ-5D-5L) (per item, range 1–5, and visual analog scale (VAS) range 0–100).
- Health appraisal: Brief Illness Perception Questionnaire short (B-IPQ) (per item, range 0–10), Rheumatology Attitudes Index (RAI) helplessness subscale (range 5–25).
- Cost-efficacy measurement by iMTA.
- Radiographic damage of the hands and feet, Sharp-van der Heijde scores (range 0–448).
- MRI-detected subclinical inflammation on the unilateral hand and foot, assessed semi-quantitatively using the RAMRIS score, evaluating synovitis (range per joint 0–3), BME (also called osteitis, range per bone 0–3), tenosynovitis (range per tendon sheath 0–3), and erosions (range per bone 0–10) [27, 28]. MRI scoring will be done with known time order and blind to any clinical data. The scores of synovitis, BME, and tenosynovitis are summed as the total MRI inflammation score (variable on the patient level). Total scores will be normalized as described by Sundin et al. [41]. Furthermore, the MRI data are categorized as “positive” and “negative” at the joint/location level and at the patient level. In addition, MRI data obtained during follow-up will also be categorized as “positive” and “negative” at the joint/location level and subsequently at the patient level. Categorization in positive/negative will be done with MRI data of symptom-free persons as a reference [13], similar to the methodology used at inclusion.
- Immunological exploratory endpoints: signatures of immune responses (such as the presence/absence of ACPA and RF, characteristics of the ACPA response, and other post-translational modifications such as anti-carbamylated protein antibodies), levels of acute-phase reactants (erythrocyte sedimentation rate, CRP), and inflammatory responses as defined through the analysis of serum and peripheral blood cell subsets (RNA expression profiling) and proteomics.
- (Serious) adverse events ((S) AEs).

### Participant timeline {13}

**Fig. 4:**
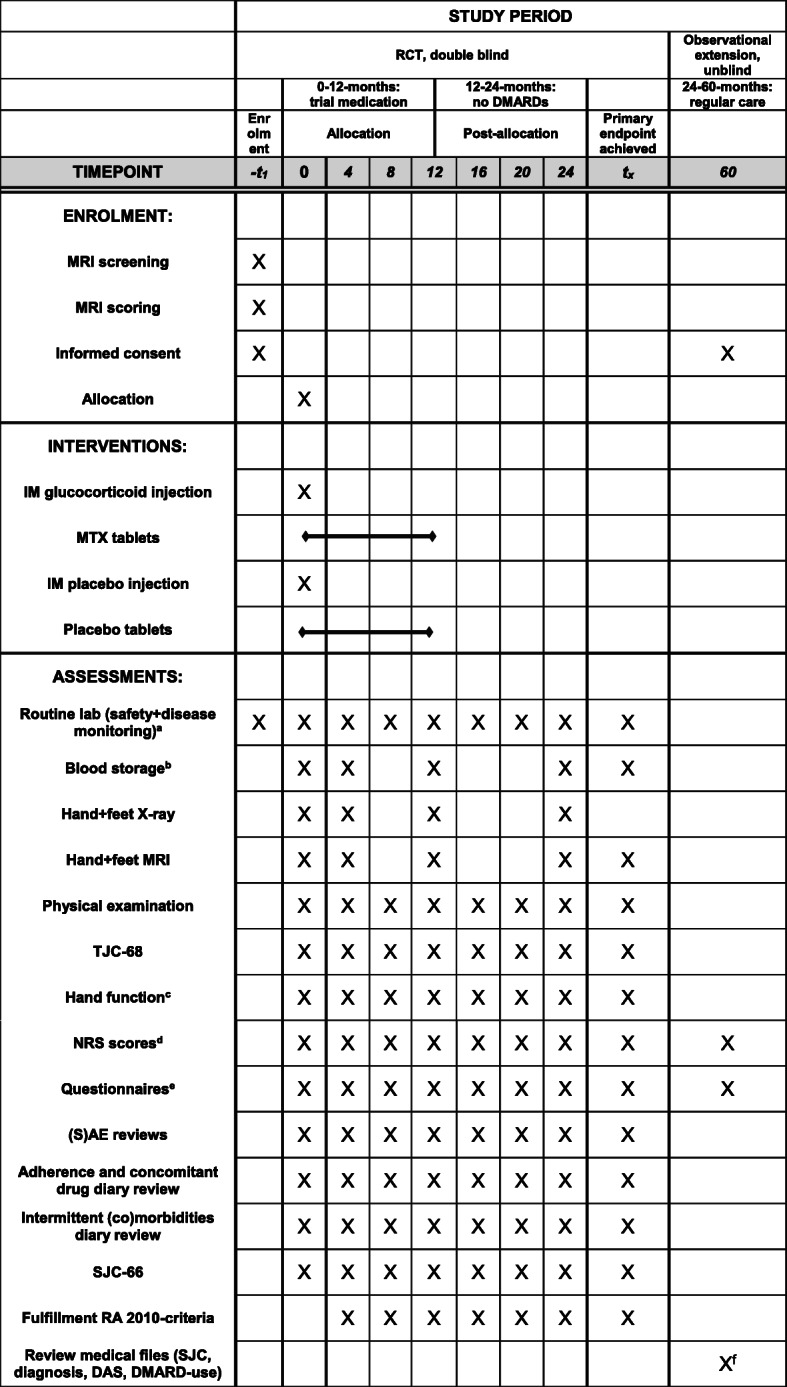
A schematic diagram of the time schedule of enrolment, intervention assessments, and visits for all participants

A schematic diagram (Fig. 4) shows the time schedule of enrolment, intervention assessments, and visits for all participants. Participants will be followed with 4-monthly intervals for 2 years within the double-blind RCT.

### Sample size {14}

Based on the results of the first 120 patients included in the longitudinal Leiden-CSA cohort and the observation that 35% of the patients with a positive MRI developed RA within 6 months of follow-up, and the assumption that treatment will result in a 50% reduction in RA development after 2 years, a sample size of 182 patients is needed to provide 80% power based on a two-sided *Z* test with pooled variance and a significance level of 0.05. Applying a 20% dropout rate results in a total sample size of 230 patients (115 per treatment arm). This dropout rate is calculated based on the data from spring 2018, when the dropout rate within the first study year was estimated at 14%. Similar sample sizes occur when calculating the time to DMARD-free status as an outcome, which is the co-primary endpoint. Based on our finding that 45% of CSA patients in the Leiden-CSA cohort had a positive MRI, we anticipate that we have to screen > 500 CSA patients to identify 230 patients with a positive MRI.

The sample size was determined for the primary endpoint. The sample size was not set to detect powered effects on the number of secondary endpoints that are assessed. The results from secondary endpoint analyses will be interpreted cautiously and in relation to the estimated confidence limits on the actual scale of the measurements. *P* value testing will be restricted, and results that are significant in isolation will be interpreted less strongly than the sets of results that are mutually supportive or that support the corresponding primary endpoint.

### Recruitment {15}

Rheumatologists working in all hospitals in the South-West region of The Netherlands have agreed to inform all eligible patients with CSA on the TREAT EARLIER trial and, with the consent of the patient, to transfer the patients’ contact details to the study doctor in the LUMC (Fig. 3). The study doctor is then responsible for the screening process.

It is known from our observational cohort data that the period of CSA with subclinical inflammation is sometimes weeks, often months, and infrequently years [14]. To prevent that patients already progress to clinical arthritis before inclusion in the trial, the logistic of screening will be organized as time-efficient and patient-friendly as possible. This means, among others, that the MRI will be scored directly after it is made. Both readers independently send their MRI scoring results to the study doctor, who compares the results with MRI data of the reference population as described in supplementary file 4. The result (positive or negativity) of the screening MRI will be communicated to the patient and if the patients prefer, and—after informed written consent—randomization can take place on the same day as the screening MRI is performed (Fig. 2).

## Assignment of interventions: allocation and blinding

### Sequence generation {16a}, concealment mechanism {16b}, and implementation {16c}

Participants will be enrolled based on a two-level definition; patients need to have arthralgia that is suspect to progress to RA according to the treating rheumatologist, and patients should have MRI-detected inflammation in the small joints (scored positive by two independent readers; both readers will be unaware of the clinical data and communicate their MRI scoring to the study doctor, without information on the scoring of the other reader). When both criteria are met and informed written consent is obtained, the study doctor, who is only in charge of the trial and does not see patients for routine care, contacts the local pharmacist of the LUMC, who will take care of the randomization, using a block randomization scheme to randomly assign the participants (no stratification will be applied). Once the participants are randomly assigned, both the IM injection and study tablets, available in the pharmacy, will be allocated to the trial participant. IM methylprednisolon or placebo injection and MTX or placebo tablets are distributed and packaged similarly. Every 4 months, study tablets will be in the same way distributed to the trial participant. All participants and staff involved in the conduct of the trial will be blind to treatment allocation throughout the trial.

### Who will be blinded {17a} and procedure for unblinding if needed {17b}

All scientific staff, rheumatologists, research nurses, and participants will remain blind to the treatment allocation. Unblinding will occur if medically necessary. In case the primary endpoint is met, participants will be treated with open-label DMARD therapy according to the routine care and the medication choice is left to the decision of the treating rheumatologist. No unblinding will take place at this time. In case participants will stop the study medication because of side effects at an earlier point in time than the 12-month period, unblinding will not take place as well (in order to prevent expectation bias influencing the treating physicians). The data safety monitoring board (DSMB) receives information blinded to the treatment allocation; however, if required for safety issues, the board can ask the pharmacist to provide them with unblinded data. Otherwise, unblinding will take place when the last study visit after 2 years of follow-up of the last included participant is done, and data quality checks (QCs), as described in the “Data management {19} and confidentiality {27}” section, are performed.

## Data collection and management

### Plans for assessment and collection of outcomes {18a}

Participants will be followed with 4-monthly intervals for 2 years. Whether there is clinically detectable arthritis will be evaluated at all these visits by physical examination of the joints by the treating rheumatologists. In case there is clinically detectable arthritis, an additional visit will be scheduled after 2 weeks to evaluate whether the arthritis is persistent (and not self-limiting) and to verify whether the primary endpoint is met. When this is the case, a visit will take place that includes all assessments, as also measured at 24 months (Fig. 4).

At every visit for 2 years, participants undergo a physical examination and hand function tests. General markers of inflammation (CRP, erythrocyte sedimentation rate), complete blood count (hemoglobin, leukocytes, thrombocytes), and liver and renal function will be measured at every study visit for 2 years. Unilateral MRIs and bilateral X-rays of the hands and feet will be made at study entry; 4, 12, and 24 months; and when the primary endpoint is achieved. Questionnaires will be completed by the participants at every study visit for 24 months.

Data collection forms can be obtained upon request by the study team (treatearlier@Lumc.nl).

Amendment (Protocol version 5, 18-01-2019) includes a deviation of MRI scan. The 1.5-T MRI scanner that is used from 2015 onwards is expected to be technically “end-of-life” after December 2019. It is foreseen that all participants are enrolled, and thus, inclusion MRI scans are completed before that time. The MRI scans made during follow-up can all be obtained either at a 1.5-T or 3-T MRI scanner, depending on the availability at the radiology department. Although in the MSK literature the performance of high-end scanners (different 1.5-T, 3-T scanners) is rather comparable [42–45], we will evaluate the comparability in detecting subclinical joint inflammation of the two scanners that we will use. Therefore, we planned to perform both 1.5-T and 3-T MRIs in up to 30 participants. This will allow us to investigate the comparability of detecting the presence of subclinical inflammation in 360 hand/foot joints, 660 bones, and 540 tendon sheaths (one scan depicts 12 small joints, 22 bones, and 18 tendon sheaths). If the presence of subclinical inflammation at the joint/bone/tendon sheath level will be similarly portrayed with both scanners, no further action will be needed. If the 3-T MRI scanner will be more/less sensitive than the 1.5-T scanner, a correction factor will be included in the analyses on the serial MRIs at the time of analyzing the results on this exploratory endpoint. This correction factor will be determined based on the data of the 30 scans that will be made on both machines. The number of participants needed to scan on both machines is difficult to calculate; however, based on the good comparability observed in the literature, the fact that we will make comparisons at the joint level, but also accounting for the fact that multiple joints/bones/tendon sheaths come from the same participant, we estimate that 30 participants are sufficient.

Both scans will be performed with at least 2 days in between to account for sufficient clearance of intravenous contrast (T1/2 of Dotarem® is 1.4–2.0 h) and with no more than 1 week in between to reduce the impact of biological variation. The US “Food and Drug Administration and the Pharmacovigilance Risk Assessment Committee” have recently concluded that no specific conditions have been linked to gadolinium contrast deposition in the organs [46]. Therefore, it is safe to administer contrast to the patients based on the current literature.

### Plans to promote participant retention and complete follow-up {18b}

The intention is to follow all participants for a total period of 24 months of the RCT at the LUMC. Participants who need to stop the study medication because of toxicity (or other reasons, e.g., wish of participant) will remain in the trial for the entire follow-up period. In case a participant finds the trial too demanding, we will ask if an adjusted scheme is an option, which will at least include the last visit at the LUMC at 24 months, and if also possible the 12-month visit.

Participants who will reach the primary endpoint will be treated by their rheumatologist according to the Dutch and international guidelines. Participants who reach the primary endpoint will be asked to complete the questionnaires at month 24.

Amendment (Protocol version 7, 25-02-2020) includes the collection of observation data between 24 and 60 months and questionnaires completed by the participants at 5 years of follow-up. All included participants will be contacted 5 years after the inclusion in the cohort to complete the questionnaires at month 60. Data from the disease course between months 24 and 60 will be retrieved from the medical files if participants consent with the observational extension study. This extension phase is submitted to and approved by the medical ethical testing committee (METC) in 2020, partly in response to comments of patient partners that suggested to determine if differences between the groups will be sustainable in the long term.

### Data management {19} and confidentiality {27}

All participant data gathered at planned and unscheduled visits will be stored and handled within the LUMC, Department of Rheumatology, in an electronic data capture (EDC) platform (ProMISe; NEN7510 certificated based on ISO27001) hosted on a dedicated secure website by the LUMC. The advanced data management (ADM) team has extensive experience with this system. Password, two-step verification management, and data exports will be controlled by the ADM team. Changes to the EDC system once the trial has begun will be minimized and will be undertaken only with the full agreement of the principal investigator, study coordinator, and the METC that is essential to the successful conduct of the study.

Participants who consent to the screening but who are subsequently found to be ineligible will also be recorded in the EDC system for “Consolidated Standards of Reporting Trials” (CONSORT) reporting purposes. These procedures will operate in accordance with the guidelines for GCP, meeting the requirements of the medicines and healthcare products regulatory agency.

Participant data will be stored under randomization number, conform to GCP guidelines. Research nurses will enter trial data in this platform, which contains study assessment data, including physical examination by a research nurse and physician data, routine lab data, (S) AE and intermittent (co)morbidities) information, and questionnaires data. The study doctor will monitor the entered data, which is a first QC, and will verify the data, checking medical forms to assure that participants have not met the endpoint and have not received concomitant medication other than NSAIDs. In addition, the study doctor will check if all (S) AEs are reported. A second QC will be performed by the study coordinator that includes all previous checks as mentioned for the study doctor. Next, the EDC system can lock every participant and every study visit, which is done by the study coordinator after approval of the principal investigator, conform to GCP guidelines.

Before unblinding (last study visit at 2 years of follow-up of the last included participant), the EDC platform will be completely locked by the ADM team under the supervision of the principal investigator. Thereafter, unblinding will take place, and the first analyses will be performed according to the statistical analysis plan.

The data manager (LUMC, Department of Rheumatology) will supervise the entire process and keep the original participant clinical forms/information/data and body material (blood) stored for at least 20 years after the last study visit of the last included participant. The ADM team, data manager, and principal investigator directly involved in this trial will be authorized to access the database but will always be blinded during the trial. In addition, QC of the data can also be performed by monitors (employees of LUMC or hired by the LUMC) and/or Dutch authorities.

All reasonable precautions to maintain the confidentiality of participants’ identities and protect the integrity of the data will be taken.

### Plans for collection, laboratory evaluation, and storage of biological specimens for genetic or molecular analysis in this trial/future use {33}

Blood samples will be processed, frozen, and stored anonymously (by randomization number) at − 80 °C locally in the LUMC for later usage. This concerns DNA, RNA, serum, and plasma. An explorative endpoint, as described in the “Outcomes {12}” section, is analyzing the signatures of immune responses and inflammatory responses.

## Statistical methods

### Statistical methods for primary and secondary outcomes {20a}

All statistical methods for the primary and secondary endpoints will be described in the statistical analysis plan, which will be completed and submitted before unblinding. Analyses will be based on the intention-to-treat (ITT) and per-protocol (PP) population. The ITT population will consist of all participants randomly assigned to a treatment group. The ITT population will form the primary analysis population of the trial and will be used for all primary and secondary efficacy endpoints and for safety analyses. This strategy is conservative because it tends to underestimate the difference in the outcome between the treatment groups, but it respects the randomization process, is free from bias, and resembles clinical practice. The PP population will consist of all participants meeting the study entry criteria, who completed follow-up and who in the first year after randomization, or up to a primary event in this period, have used ≥ 8 study tablets a week (equivalent to at least 20 mg/week MTX) for 80% of the time and used no forbidden medication (no systemic or intra-articular steroids or DMARDs outside the study protocol) during the entire follow-up period. An MTX dose of ≥ 20 mg/week has been shown to provide optimal efficacy in RA [31, 32, 34]; therefore, a dose of ≥ 8 study tablets was incorporated in the definition. The PP population will consist of participants who strictly adhere to the protocol and provide an estimate of the true efficacy of the MTX intervention. All primary and secondary efficacy endpoints will also be analyzed in the PP population. The primary analysis will be the time to the development of the primary endpoint. The co-primary endpoint is the difference in the percentage of patients that achieved DMARD-free status at 24 months. Significance will be assessed by a gate-keeping analysis. We do prioritize the two primary endpoints by testing the primary endpoint first, and only if this test is significant, the co-primary endpoint will be tested.

### Interim analyses {21b}, methods for additional analyses (e.g., subgroup analyses) {20b}, methods in analysis to handle protocol non-adherence, and any statistical methods to handle missing data {20c}

No interim analyses will be performed during the trial. Additional analyses, analysis to handle protocol non-adherence, and any statistical methods to handle missing data will be described in the statistical analysis plan.

### Plans to give access to the full protocol, participant-level data, and statistical code {31c}

The full protocol, participant-level data, and statistical codes will be available from the corresponding author upon reasonable request.

## Oversight and monitoring

### Composition of the coordinating center and trial steering committee {5d} and composition of the data monitoring committee, its role, and reporting structure {21a}

This trial will be the responsibility of the LUMC and performed at the LUMC and is thus the coordinating center. For the roles of everyone involved in this trial within the LUMC, we refer to the supplementary file 6.

Data monitoring will be performed by the DSMB that is independent of the principal investigator and has no competing interests: for further details on composition, its role, and reporting structure, we refer to the charter in supplementary file 7.

### Adverse event reporting and harms {22}

AEs are clinically significant changes in vital signs, laboratory test abnormalities, and clinical tolerability of the study medication. An AE is any adverse medical change from the subject’s baseline (or pre-treatment) condition which occurs during the course of a clinical study, after starting treatment, whether considered treatment-related or not. AEs may be mentioned spontaneously by the participant, or be discovered as a result of general questioning by the rheumatologist or by physical examination. AEs will be monitored at each follow-up visit by asking the participant open-ended questions to identify any problems that have occurred since the previous visit. The treating rheumatologist will decide if and how action should be taken as a response to an AE. The toxicities to be expected of MTX and a single injection of methylprednisolon are all well known to the participating rheumatologists and generally mild. The research nurse or study doctor will record the AE(s) in the clinical record file. As much as possible, each AE must also be described by its duration (start and end dates), its frequency (single episode, intermittent, continuous), its severity (mild, moderate, severe), an assessment of its cause (the underlying study indication, coexisting disease, concomitant medication, the study medication, or others), its relationship to the study medication (unrelated, unlikely, possibly, probably, definitely), whether it influenced the course of the study medication, or whether it required specific therapy.

A serious AE (SAE) is any event that is fatal or life-threatening, is permanently or significantly disabling, requires or prolongs hospitalization, involves cancer or congenital anomaly, or occurs with overdose, either intentional or inadvertent. All SAEs, whether or not deemed drug related, will be reported directly and communicated to the therefore appropriate authority (toetsingonline.nl and METC).

The DSMB, consisting of three independent physicians of the LUMC, is able to make trial decisions regarding major issues like (unexpected) toxicity. All SAE reports will also be presented to the DSMB.

### Frequency and plans for auditing trial conduct {23}

There are no planned auditing trial conducts.

### Plans for communicating important protocol amendments to relevant parties (e.g., trial participants, ethical committees) {25}

Protocol amendments will be communicated to the local Leiden Den Haag Delft–METC for approval. After ethical approval, trial participants, registries, and trial staff will be updated on the protocol modifications depending on the influence of the changes this could be by phone, e-mail, face-to-face meetings, and/or newsletters.

### Dissemination plans {31a}

Trial results will be communicated to the participants by a symposium and newsletters. Trial results will also be open access reported via publication. We also intend to inform the Dutch public by a release of the result by ReumaNederland.

## Discussion

This protocol describes the TREAT EARLIER trial that aims to achieve secondary prevention of the most common form of inflammatory arthritis, namely RA. It studies the efficacy of the first-line therapy of RA (MTX, in combination with methylprednisolon) that is normally started in the phase of clinically apparent arthritis and is now evaluated in the phase of CSA with subclinical inflammation. MTX is the anchor drug in RA because it is efficacious, safe, cheap ,and is also the basis for combination therapies (for instance with glucocorticoids). According to international guidelines, MTX is typically escalated in 4–6 weeks’ time to 20–25 mg/week [33]. Despite the widespread use and known clinical efficacy, the mechanism by which MTX (in the described dose) exerts its effect is incompletely understood. Although peak plasma concentrations are generally reached within hours after administration and it has disappeared from the circulation in approximately 24 h, a time lag occurs before a clinical benefit is seen. MTX is known for its slow onset of action, and therefore, in this trial, a single IM injection of corticosteroids was added at treatment start for a quicker action. A number of mechanisms are identified that are potentially involved in the efficacy of MTX, among which is the depletion of metabolite levels that reduce the replication or survival of lymphocytes or pathogenic cell types. In addition to the antagonism of folate-dependent processes, stimulation of adenosine signaling (e.g., including FOXP3+ Treg cells), generation of reactive oxygen species, and downregulation of adhesion molecule expression and of matrix metalloproteinases, as wells as the modification of cytokine profiles, have been described [47]. MTX therefore has pleiotropic effects that could be important in modifying disease processes underlying the progression from arthralgia with subclinical inflammation to persistent clinical arthritis.

The duration of DMARD use of 1 year in this proof-of-concept trial is empirical. If it is efficacious, further studies are needed to determine the optimal dosage and duration of treatment that is required to achieve permanent disease modification.

The follow-up duration of 24 months seems sufficient for the assessment for the primary endpoint, because our present data suggest that the majority of CSA patients who convert to clinically detectable arthritis and RA do so within 4–6 months [14]. The follow-up includes 12 months without study medication. Supposed that MTX would postpone but not prevent the development of RA, a medication-free period of 12 months should be sufficient to detect this. Also, in the PROMPT trial, only two patients developed RA later than 12 months after cessation of study medication [48]. Nonetheless, the most recently approved amendment to the protocol (approved February 2020, protocol version 7) includes an observational extension of the trial with a total follow-up time of 5 years, as the achievement of an outcome after a relatively short time period of time is not reflective of the subsequent disease course [49]. A long-term follow-up is required to evaluate whether results are sustainable and valuable. Especially, patients have indicated that the current disease burden is mostly caused by pain, fatigue, and functional impairments [50]; the decision to extend the follow-up duration is therefore in line with the suggestions of patient partners. After 5 years follow-up, we will assess primary and secondary endpoints, to evaluate if differences are permanent in the longer term.

The primary endpoint is assessed by physical examination. Clinical assessments are to some extent prone to interindividual variation especially when no efforts are undertaken to promote comparability. Therefore, reliability sessions have been held at the start of the trial with all participating rheumatologists. At these sessions, patients with a broad range of disease activity, ranging from zero to many swollen joints, were present and scored by all rheumatologists. Although some variation occurred in the number of swollen joints scored in few patients with very active RA (these patients had many tender and swollen joints), the presence or absence of swollen joints (clinical arthritis) at the patient level was very reproducible scored by all rheumatologists. In addition, the endpoint joint swelling is observed by two independent rheumatologists; this is done to reduce interobserver variation and is also considered valuable when rheumatologists in training are the treating rheumatologist. Furthermore, to verify that the endpoint, joint swelling, was not subtle, the RA criteria [22] should be fulfilled or clinical arthritis should be clearly present in at least 2 joints at physical examination and without having information of imaging modalities. In addition, to further ensure that synovitis is persisting, the joint swelling (clinical arthritis) should remain to be present after an interval of 2 weeks. Although a period longer than 2 weeks would be even better to assess persistency, delaying treatment with DMARDs for more than 2 weeks in the presence of clinical arthritis was considered inappropriate by the treating rheumatologists. All the mentioned measures were taken to have a robust assessment of the endpoint clinical arthritis.

The primary endpoint can be considered as the main outcome assessed from the perspective of rheumatologists, but patients indicated that the burden of RA is best expressed by assessing the domains of independence (functioning, workability), pain, and fatigue [50]. The secondary endpoints may therefore be considered as the benefit from very early treatment from patients’ perspectives. Moreover, an evaluation 1 year after the cessation of study medication may be insufficient to determine if favorable effects are sustainable over time [49]. Thus, as PROs are considered most valuable by patients, and as the 2 years measurement insufficiently assesses sustainability, all secondary endpoints will also be analyzed again 5 years after the study start.

This trial was designed in 2014, and risk stratification was based on the first 120 included Leiden-CSA patients, which resulted in a risk of 35% to progress to RA during the next year (PPV) in patients with CSA and a positive MRI. Based on these numbers, a sample size of 230 participants was required for a well-powered study. Recently, it was observed that higher PPVs can be obtained when weighting tenosynovitis (particularly, MCP extensor peritendinitis) and the number of locations with subclinical inflammation (PPVs up to 63–67%) [17]. This creates opportunities for (subgroup) analyses on participants with high-risk characteristics.

CSA patients will be enrolled in this trial irrespective of the auto-antibody status as it has been reported that with current treatment strategies, the disease burden is similar in ACPA-positive and ACPA-negative RA patients [19], and in addition, EULAR recommendations for the management of early arthritis recommend MTX as the first-line therapy, independent of the auto-antibody status [20]. Nevertheless, there is accumulating evidence that ACPA-positive and ACPA-negative RA have different etiopathologies, as underlying genetic [51] and environmental risk factors differ [52, 53]. Also, subtle differences in symptoms [54] and timelines of the symptomatic phase that precedes the development of clinical arthritis have been reported [55]. It is therefore possible that disease maturation in the symptomatic pre-arthritis phase differs between the two groups. Consequently, the influence of treatment may be different in APCA-positive and ACPA-negative CSA. In addition to adding an adjustment factor, this can be addressed by stratifying the analyses for ACPA.

Evidence indicating whether or not intervention in the pre-arthritis phase of CSA reduces disease persistence will be an important step forward, both for individual patients and for society. In case very early intervention with a cheap medication such as MTX will be effective, this will most likely concomitantly reduce the total level of pain, the level of functional loss, and the duration of symptoms. Because of the impact of RA on the lives of individual patients, such outcomes will be highly relevant for patients and may entail a considerable decrease in RA-related costs for society. A cost-effectiveness analysis will be performed at the end of the study.

The results of this proof-of-concept study will be relevant for science. Current research is focused on identifying essential processes that occur during the window of opportunity. This is done by association studies, evaluating potential risk factors, such as the presence of inflammatory markers, auto-antibody responses, and imaging features, in patients in different phases of disease development. These association studies will be brought to the next level by a placebo-controlled intervention study. Studies on the bio-samples that will be obtained during the trial will increase the comprehension of the inflammatory and autoimmune processes relevant for disease “chronification.” At present, it is not yet known whether interventions in patients at risk for RA are effective. The results of this study are positive in case treatment prevents the development of RA or reduces disease persistency. In case of positive findings, further trials on optimal treatment strategies are required to allow optimized implementation.

## Trial status and other additional information

The TREAT EARLIER study trial received ethical approval on 2 March 2015 (Protocol version 2, 12-01-2015). The trial is registered at Dutch Trial Register (NTR4853/Trial NL4599, https://www.trialregister.nl/trial/4599); 20 October 2014.

The first participant was randomly assigned in April 2015. The RCT is expected to end in September 2021.

- Trial-start: Protocol version 1 (20-10-2014) changes were required, and version 2 (12-01-2015) was approved.
- First amendment. Increase samples from 200 to 230 due to expected larger dropout rate than included in first sample size calculation (20% versus 10%): protocol version 3 (26-03-2018) changes were required, and version 4 (31-05-2018) was approved.
- Second amendment. Deviation of MRI scan as the MRI scanner that is used from 2015 onwards is expected to be technically “end-of-life” by December 2019: protocol version 5 (18-01-2019) was approved. Up to 30 participants will be scanned on 1.5-T and 3-T MRI.
- Third amendment includes a collection of observational data between 24 and 60 months: protocol version 6 (03-02-2020) changes were required, and version 7 (25-02-2020) was approved.

## Supplementary information

**Additional file 1: Supplementary file 1.** WHO trial registration Data Set (Version 1.3). **Supplementary file 2.** Referring centers in the South-West region of The Netherlands. **Supplementary file 3.** MRI scan protocol. **Supplementary file 4.** MRI scan evaluation. **Supplementary file 5.** ICCs obtained after extensive training period of all readers. **Supplementary file 6.** Composition of the coordinating centre. **Supplementary file 7.** Charter of DSMB. **Supplementary file 8.** Example of informed consent form of the TREAT EARLIER trial.

## Abbreviations

ACPA: Anti-citrullinated peptide antibodies
ADM: Advanced data management
AE: Adverse event
B-IPQ: Brief Illness Perception Questionnaire
BME: Bone marrow edema
CSA: Clinically suspect arthralgia
CONSORT: Consolidated Standards of Reporting Trials
CRP: C-reactive protein
DMARD: Disease-modifying anti-rheumatic drug
DSMB: Data safety monitoring board
EDC: Electronic data capture
ETL: Echo train length
EULAR: European League Against Rheumatism
EQ-5D-5L: EuroQol 5D-5level
Fatsat: Fat saturation
FSE: Fast spin-echo
GCP: Good Clinical Practice
HAQ-DI: Health Assessment Questionnaire Disability Index
ICC: Intraclass correlation coefficient
ITT: Intention-to-treat
IM: Intramuscular
LUMC: Leiden University Medical Center
MCP: Metacarpophalangeal
METC: Medical ethical testing committee
MRI: Magnetic resonance imaging
MSK: Musculoskeletal
MTP: Metatarsophalangeal
MTX: Methotrexate
NRS: Numeric rating scale
NSAID: Non-steroidal anti-inflammatory drug
OMERACT: Outcome Measures in Rheumatology Clinical Trials
PP: Per-protocol
PRO: Patient-reported outcome
QC: Quality check
RA: Rheumatoid arthritis
RAI: Rheumatology Attitudes Index
RAMRIS: RA MRI scoring
RCT: Randomized-controlled trial
RF: Rheumatoid factor
RMD: Rheumatic and musculoskeletal disease
SAE: Serious adverse event
SF-36: Short Form-36
SJC: Swollen joint count
STIR: Short tau inversion recovery
TE: Echo time
TJC: Tender joint count
TR: Repetition time
VAS: Visual analog scale
WPAI: Work productivity and impairment scale
