# Supplementary material for: TREAT Early Arthralgia to Reverse or Limit Impending Exacerbation to Rheumatoid arthritis (TREAT EARLIER): a randomized, double-blind, placebo-controlled clinical trial protocol
Source: Trials. 2020 Oct 16;21:862. doi: 10.1186/s13063-020-04731-2 (PMC7574479; doi:10.1186/s13063-020-04731-2)
Supplement: Supplementary file 1 — Additional file 1: Supplementary file 1. WHO trial registration Data Set (Version 1.3). Supplementary file 2. Referring centers in the South-West region of The Netherlands. Supplementary file 3. MRI scan protocol. Supplementary file 4. MRI scan evaluation. Supplementary file 5. ICCs obtained after extensive training period of all readers. Supplementary file 6. Composition of the coordinating centre. Supplementary file 7. Charter of DSMB. Supplementary file 8. Example of informed consent form of the TREAT EARLIER trial. [file 13063_2020_4731_MOESM1_ESM.docx]

**Supplementary file 1**

**WHO trial registration Data Set (Version 1.3)**

| 1. Primary registration and trial identifying number | Dutch Trial Register, Trial NL4599 |
| --- | --- |
| 1. Date of Registration in primary registry | 20^th^ of October 2014 |
| 1. Secondary identifying numbers | Trial register: NTR4853  EudraCT: NL2014-004472-35  ABR/Toetsing online: NL51205.058.14 |
| 1. Source(s) of monetary or material support | This trial is financially supported by a ZonMW grant (programma translationeel onderzoek). The remaining costs will be covered by the department of rheumatology of the Leiden University Medical Center |
| 1. Primary sponsor | Leiden University Medical Center (non-commercial) |
| 1. Secondary sponsor(s) | Not applicable |
| 1. Contact for public queries | PI: Prof. dr. A.H.M. van der Helm-van Mil; [A.H.M.van_der_Helm@lumc.nl](mailto:A.H.M.van_der_Helm@lumc.nl) or +31 71 526 3598, Albinusdreef 2, 2333 ZA Leiden, The Netherlands |
| 1. Contact for scientific queries | PI: Prof. dr. A.H.M. van der Helm-van Mil, [A.H.M.van_der_Helm@lumc.nl](mailto:A.H.M.van_der_Helm@lumc.nl) or +31 71 526 3598, Department of Rheumatology, Leiden University Medical Center, Leiden, Albinusdreef 2, 2333 ZA Leiden, The Netherlands |
| 1. Public title | TREAT EARLIER |
| 1. Scientific title | Treat early arthralgia to reverse or limit impending exacerbation to rheumatoid arthritis (TREAT EARLIER) |
| 1. Countries of recruitment | The Netherlands |
| 1. Health condition(s) or problem(s) studied | Arthralgia |
| 1. Interventions | Intervention arm: one intramuscular methylprednisolone injection and 1-year of methotrexate tablets (25mg/week)  Control arm: one intramuscular placebo injection and 1-year of placebo tablets |
| 1. Key inclusion and exclusion criteria | Inclusion criteria:  1. Age ≥18 years.  2. Patients without clinically detectable arthritis but with arthralgia of small hand or feet joints of recent-onset (<1-year) that according to the rheumatologist is suspect to be an early presentation of RA (this symptom complex is called CSA).  3. Unilateral MRI of hand and foot joints, positive for subclinical inflammation.  4. Ability and willingness to give written informed.  Exclusion criteria:  1. Symptoms or signs making diagnoses other than RA more likely. These are amongst others; >6 tender points or Heberden or Bouchard nodules (the presence of such characteristics precludes CSA).  2. Presence of, or history of, clinically apparent arthritis (this precludes CSA).  3. Previous or current treatment with DMARDs or corticosteroids (this precludes CSA).  4. Contra indications for MRI: certain metal implants, pacemakers, GFR < 30ml/min.  5. Pregnancy or the wish to become pregnant, breast feeding.  6. Bone marrow hypoplasia.  7. Elevated hepatic enzyme levels (ASAT, ALAT >3 times normal value).  8. Serum creatinine level >150umol/l or estimated clearance of <60%.  9. Serious infections such as hepatitis, pyelonephritis in the past three months, or chronic infectious disease such as chronic chest infections with bronchiectasis. |
| 1. Study type | Intervention, randomized, double-blinded, parallel, phase II (therapeutic exploratory) trial |
| 1. Date of first enrollment | 16^th^ of April 2015 |
| 1. Sample size | Target number of participants: 230  Included number of participants: 236 |
| 1. Recruitment status | Complete |
| 1. Primary outcome(s) | Primary outcome  Name: clinically detectable arthritis  Method of measurement: either fulfilling the 2010 criteria for RA or unclassified arthritis with ≥2 swollen joints at physical joint examination confirmed by two rheumatologists that persists for at least 2-weeks  Timepoint: during a 2-year follow-up period  Co-primary outcome  Name: DMARD-free status  Method of measurement: absence of clinically detectable synovitis at joint examination in the absence of DMARD-use (including systemic or intra-articular glucocorticoids).  Timepoint: at the 2-year follow-up |
| 1. Key secondary outcomes | Name: patient reported outcomes  Method of measurement: Health Assessment Questionnaire Disability Index (HAQ-DI), Work Productivity And Impairment scale (WPAI), pain number rating score (NRS), fatigue NRS, morning stiffness NRS and duration.  Timepoint: every 4-months for 2-years |
| 1. Ethics review | Approved by local Leiden Den Haag Delft – medical ethical testing committee (METC, Postbus 9600, 2300 RC Leiden, The Netherlands, metc-ldd@lumc.nl) at 2^nd^ of March 2015 |
| 1. Completion date | September 2021 |
| 1. Summary results | Not applicable, study is ongoing |
| 1. IPD sharing statement | Undecided if individual clinical trial participant-level data (IPD) will be shared |

**Supplementary file 2**

**Referring centers in the South-West region of The Netherlands**

Albert Schweitzer

Alrijne Hospital

Erasmus Medical Center

Franciscus Gasthuis & Vlietland

Groene Hart Hospital

Haaglanden Medical Center Antoniushoeve

Haaglanden Medical Center Bronovo

Haaglanden Medical Center Westeinde

Haga Hospital

Haven-policlinic Rotterdam

IJselland Hospital

Ikazia Hospital

Langeland Hospital

Meander Medical Center

Maasstad Hospital

Reinier de Graaf Gasthuis

Reumazorg Zuid-West Nederland

Spaarne Gasthuis

**Supplementary file 3**

**MRI scan protocol**

In the hand (metacarpophalangeal (MCP)2-5 and wrist) the following sequence will be acquired before contrast administration: T1-weighted fast spin-echo (FSE) sequence in the coronal plane (repetition time (TR) 575ms, echo time (TE) 11.2ms, acquisition matrix 388×288, echo train length (ETL) 2). After intravenous injection of gadolinium contrast (gadoteric acid (Dotarem), Guerbet, Paris, France, standard dose of 0.1mmol/kg) the following sequences will be obtained: T1-weighted FSE sequence with frequency selective fat saturation (fatsat) in the coronal plane (TR/TE 700/9.7ms, acquisition matrix 364×224, ETL 2), T1-weighted FSE fatsat sequence in the axial plane (wrist: TR/TE 540/7.7ms; acquisition matrix 320x192; ETL 2 and MCP-joints: TR/TE 570/7.7ms; acquisition matrix 320x192; ETL 2). The obtained post-contrast sequences of the foot (metatarsophalangeal (MTP)1-5 joints) will be: T1-weighted FSE fatsat sequence in the axial plane (TR/TE 700/9.5ms; acquisition matrix 364x224, ETL 2) and T1-weighted FSE fatsat sequence in the coronal plane (perpendicular to the axis of the metatarsals) (TR/TE 540/7.5ms; acquisition matrix 320x192, ETL 2).

Patients will be asked not to use any non-steroidal anti-inflammatory drugs (NSAIDs) during the 24-hours before MRI. The scan protocol is almost similar to the protocol recommended by the ‘outcome measures in rheumatology clinical trials’ (OMERACT)-rheumatoid arthritis (RA) MRI scoring (RAMRIS) study group.(1) We use the contrast-enhanced T1-weighted fat suppressed sequence to assess bone marrow edema (BME) in the MCP- and MTP-joints of all patients. According to the RAMRIS-method, T2-weighted fat suppressed sequences, or when this sequence is not available a short tau inversion recovery (STIR) sequence, should be used to assess BME. However, three previous studies have demonstrated that a contrast-enhanced T1-weigthed fat suppressed sequence has a strong correlation with T2-weighted fat suppressed sequences.(2-4) Furthermore, the arthritis subcommittee of the ‘European society of musculoskeletal radiology’ also recommends the use of contrast-enhanced T1-weighted fat suppressed sequences for depicting BME.(5) The T2-weighted image shows increased water signal and a contrast-enhanced T1-weighted sequence shows increased water content and the increased perfusion and interstitial leakage. A strong correlation has been shown in arthritis patients and in patients without inflammatory diseases such as bone bruises, intraosseous ganglions, bone infarcts and even nonspecific cases.(3, 4) Thus, although T2-weighted frequency selective fatsat or STIR images, and gadolinium-chelate-enhanced T1-weighted fast spin echo sequences with frequency-selective fatsat have different underlying causes for the signal intensity (technical considerations are recently reviewed in (6)), the sequences perform equally in depicting BME. Therefore, BME will be assessed on contrast-enhanced T1-weighted fat suppressed sequences as it has a higher signal to noise ratio and allows a shorter scan time for patients. In addition, because T2-weighted fat suppressed sequences can be omitted, coronal sequences of the foot can be added. In total this results in a shorter total scan time (on average 60mins) and more information.

Field-of-view is 100mm for the hand and 140mm for the foot. Coronal sequences of the hand have 18 slices with a slice thickness of 2mm and a slice gap of 0.2mm. Coronal sequences of the foot have 20 slices with a slice thickness of 3mm and a slice gap of 0.3mm. All axial sequences have a slice thickness of 3mm and a slice gap of 0.3mm with 20 slices for the wrist, 16 for the MCP-joints and 14 for the foot.

**Supplementary file 4**

**MRI scan evaluation**

Synovitis will be assessed, ranging 0-3, in 12 joints: MCP2-5, MTP1-5, and in three regions of the wrist. The total synovitis score has a range of 0-36. Subclinical inflammation will be assessed in these 12 joints and counted, resulting in a score of 0-12 locations of MRI-detected inflammation due to synovitis.

BME will be scored, ranging 0-3, in the proximal and distal MCP2-5 (8 bones) and, proximal and distal MTP1-5 (10 bones) separately, and in 13 bones in the wrist. In the wrist the carpometacarpal-1 joint (base of meta-carpal 1 and the trapezium) will be excluded, since these are considered osteoarthritis locations.(7) The proximal and distal bones of MCP- and MTPs will be summed per joint. Therefore, 22 locations will be assessed for BME: 4 for the MCPs, 5 for the MTPs (both with a range of 0-6 per joint) and 13 for the wrist (with a range of 0-3 per bone). The total BME score will range from 0-93. Tenosynovitis will be scored, ranging 0-3, in 18 tendons: 10 tendons in the wrist and 8 for the MCPs (4 flexor and 4 extensor tendons). Thus, the total tenosynovitis score will be a sum of 18 tendons and ranging 0-54.

The number of locations of subclinical inflammation due to synovitis, BME, and tenosynovitis will be summed, resulting in a number of locations of subclinical inflammation ranging 0-52. Total synovitis, BME, and tenosynovitis scores will be summed into the total inflammation score, ranging 0-183.

Per location, scores of synovitis, tenosynovitis, and BME will be compared to the scores of healthy controls at the same location in the matching age-group (<40, 40-60, and >60 years old). A location will be considered positive if each of the two readers independently indicates presence of the inflammatory lesion, and in addition this lesion is present in <5% of the healthy persons in the same age-category.(7-9) An MRI will be considered positive if a patient has at least one positive lesion as described. If only one reader identifies the presence of an inflammatory lesion (that is at the same location present in <5% of the healthy persons in the same age-category) the MRI will be considered negative.

**Supplementary file 5**

**ICCs obtained after extensive training period of all readers**

**Interreader reliability**

|  | **1** | **2** | **3** | **4** | **5** | **6** | **7** | **8** | **9** | **10** |
| --- | --- | --- | --- | --- | --- | --- | --- | --- | --- | --- |
| **1** | x | 0.97 | 0.97 | 0.98 | 0.97 | 0.96 | 0.95 | 0.97 | 0.93 | 0.96 |
| **2** | 0.97 | x | 0.99 | 0.95 | 0.94 | 0.95 | 0.94 | 0.96 | 0.93 | 0.94 |
| **3** | 0.97 | 0.99 | x | 0.95 | 0.95 | 0.95 | 0.96 | 0.96 | 0.94 | 0.96 |
| **4** | 0.98 | 0.95 | 0.95 | x | 0.97 | 0.96 | 0.94 | 0.95 | 0.91 | 0.95 |
| **5** | 0.97 | 0.94 | 0.95 | 0.97 | x | 0.95 | 0.94 | 0.95 | 0.92 | 0.93 |
| **6** | 0.97 | 0.95 | 0.95 | 0.96 | 0.95 | x | 0.95 | 0.96 | 0.95 | 0.96 |
| **7** | 0.95 | 0.94 | 0.96 | 0.94 | 0.94 | 0.95 | x | 0.98 | 0.98 | 0.97 |
| **8** | 0.97 | 0.96 | 0.96 | 0.95 | 0.95 | 0.96 | 0.98 | x | 0.96 | 0.96 |
| **9** | 0.93 | 0.93 | 0.94 | 0.91 | 0.92 | 0.95 | 0.98 | 0.96 | x | 0.97 |
| **10** | 0.96 | 0.94 | 0.96 | 0.95 | 0.93 | 0.96 | 0.97 | 0.96 | 0.97 | x |

**Intrareader reliability**

| **1** | **2** | **3** | **4** | **5** | **6** | **7** | **8** | **9** | **10** |
| --- | --- | --- | --- | --- | --- | --- | --- | --- | --- |
| 0.99 | 0.98 | 0.94 | 0.92 | 0.96 | 0.94 | 0.98 | 0.99 | 0.96 | 0.98 |

**Supplementary file 6**

**Composition of the coordinating centre**

**Principal investigator**
Prof. dr. A.H.M. van der Helm-van Mil (rheumatologist, Department of Rheumatology, LUMC)

**Study-coordinator**

Dr. E. Niemantsverdriet (postdoc/coordinator, Department of Rheumatology, LUMC)

**Study-doctors**

Dr. L.E. Burgers (PhD student, Department of Rheumatology, LUMC, 2015-2018)

Drs. Y.J. Dakkak (PhD student, Department of Rheumatology, LUMC, 2017-2020)

Drs. D.I. Krijbolder (PhD student, Department of Rheumatology, LUMC, 2020-current)

**Project advisors**

Dr. C.F. Allaart (internist, Department of Rheumatology, LUMC)

Prof. dr. T.W.J. Huizinga (head of Department of Rheumatology, LUMC)

Dr. M. Reijnierse (head of Department of Radiology, LUMC)

Prof. dr. A.A. Kaptein (advisor, Department of Medical psychology, LUMC)

Prof. dr. A. Boonen (rheumatologist, Department of Rheumatology, UMC Maastricht)

**Gebruikerscommisie**

ZonMw Delegate

ReumaNederland delegate: Mw. N. Klomp or I. Lether

Dr. C.F. Allaart (internist, Department of Rheumatology, LUMC)

Mw. L. de Smet (patient CSA/RA)

Mw. J.W. Blom (general practitioner)

Prof. dr. A.H.M. van der Helm-van Mil (chair; rheumatologist, Department of Rheumatology, LUMC)

**DSMB**

Prof. dr. R. ten Cate (Willem-Alexander Children Hospital, Department of pediatric rheumatology, LUMC)

Prof. dr. S. le Cessie (Department of Epidemiology, LUMC)

Dr. A.M.J. Langers (Department of Gastroenterology and Hepatology, LUMC)

**Data management**

Ms. J.W.M. Krol-van Berkel (Department of Rheumatology, LUMC)

**Statisitical support**

Prof. dr. E.W. Steyerberg (Department of Medical Statistics and Bioinformatics, LUMC)

Dr. S. Böhringer (Department of Medical Statistics and Bioinformatics, LUMC)

**Coordinating centre:**

Leiden University Medical Center

Department of Rheumatology, C4-R

P.O. Box 9600

2300 RC Leiden, The Netherlands

Telephone: (+31) 071-5263592 / 3598

**Supplementary file 7**

**Charter of DSMB**

##

| Content |  |
| --- | --- |
| 1. Introduction |  |
| Name (and sponsor’s ID) of trial plus ISRCTN and/or EUDRACT number | **TREAT EARLY ARTHRALGIA TO REVERSE OR LIMIT IMPENDING EXACERBATION TO RHEUMATOID ARTHRITIS**  **Acronym: TREAT EARLIER**  2014-004472-35 |
| Objectives of trial, including interventions being investigated | This proof-of-concept study aims to determine whether intervention in the preclinical phase in symptomatic patients at risk for RA is effective in progression from subclinical inflammation to clinically apparent persistent arthritis |
| Outline of scope of charter | The purpose of this document is to describe the roles and responsibilities of the independent DMC for the TREAT EARLIER trial. |
| 2. Roles and responsibilities |  |
| A broad statement of the aims of the committee | To safeguard the interests of trial participants, assess the safety and efficacy of the interventions during the trial, and monitor the overall conduct of the clinical trial. |
| Terms of reference | The DMC should receive and review the progress and accruing data of this trial and provide advice on the conduct of the trial to the Trial Steering Committee.  The DMC should inform the Chair of the steering committee if, in their view:  (i) the results are likely to convince a broad range of clinicians, including those supporting the trial and the general clinical community, that one trial arm is clearly indicated or contraindicated, and there was a reasonable expectation that this new evidence would materially influence patient management; **or**  (ii) it becomes evident that no clear outcome would be obtained. |
| Specific roles of DMC | Interim review of the trial’s progress including updated figures on recruitment, data quality, and main outcomes and safety data.  A selection of specific aspects could be compiled from the following list:-   - monitor recruitment figures and losses to follow-up - monitor evidence for treatment harm (eg toxicity data, SAEs, deaths) - decide whether to recommend that the trial continues to recruit participants or whether recruitment should be terminated either for everyone or for some treatment groups and/or some participant subgroups - advise on protocol modifications suggested by investigators or sponsors (eg to inclusion criteria, trial endpoints, or sample size) - considering the ethical implications of any recommendations made by the DMC |
| 3. Before or early in the trial |  |
| Whether the DMC will have input into the protocol | All DMC members have seen the protocol before agreeing to join the committee. |
| Whether the DMC will meet before the start of the trial | no |
| Any issues specific to the disease under study | na |
| Any specific regulatory issues | na |
| Any other issues specific to the treatment under study | na |
| Whether members of the DMC will have a contract | no |
| 4. Composition |  |
| Membership and size of the DMC | Prof. dr. S. le Cessie (Department of Epidemiology, LUMC)  Dr. A.M.J. Langers (Department of Gastroenterology and Hepatology, LUMC)  Prof. dr. R. ten Cate (Willem-Alexander Children Hospital, LUMC) |
| The Chair, how they are chosen and the Chair’s role. (Likewise, if relevant, the vice-Chairman) | Prof. dr. R. ten Cate (Willem-Alexander Children Hospital, LUMC) |
| 5. Relationships |  |
| Relationships with Principal Investigators, other trial committees (eg Trial Steering Committee (TSC) or Executive Committee), sponsor and regulatory bodies | The DMC advices the Trial steering committee. |
| Clarification of whether the DMC are advisory (make recommendations) or executive (make decisions) | The DMC does not make decisions about the trial, but rather makes recommendations to the steering committee. |
| Payments to DMC members | None |
| The need for DMC members to disclose information about any competing interests | Competing interests will be disclosed. |
| 6. Organisation of DMC meetings |  |
| Expected frequency of DMC meetings | The DMC will get a 4 monthly overview of inclusions and SAEs by email, and subsequently act upon that if required. |
| Whether meetings will be face-to-face or by teleconference | The meetings are held by mail and if required or preferred by the DMC (e.g. based on the issues to be discussed) in person (face to face). |
| How DMC meetings will be organised, especially regarding open and closed sessions, including who will be present in each session | The meeting will have a mixture of open and closed sessions. |
| 7. Trial documentation and procedures to ensure confidentiality and proper communication |  |
| Intended content of material to be available in open sessions | Accumulating information relating to recruitment will be presented. Toxicity details based on pooled data will be presented and total numbers of events for the primary outcome measure and other outcome measures may be presented, at the discretion of the DMC. |
| Intended content of material to be available in closed sessions |  |
| Will the DMC be blinded to the treatment allocation | Yes |
| Who will see the accumulating data and interim analysis | There will be no interim analysis |
| Who will be responsible for identifying and circulating external evidence (eg from other trials/ systematic reviews) | Identification and circulation of external evidence (eg from other trials/ systematic reviews) is not the responsibility of the DMC members. The PI (Dr Van der Helm) will collate any such information. |
| To whom the DMC will communicate the decisions/ recommendations that are reached | The DMC will report its recommendations to the Trial Steering Committee. |
| Whether reports to the DMC be available before the meeting or only at/during the meeting | For meetings per mail this is no issue, for the meetings in person the DMC will receive the required information at least a week before the meeting. |
| What will happen to the confidential papers after the meeting | The DMC members should store the papers safely after each meeting so they may check the next report against them. After the trial is reported, the DMC members should destroy all interim reports. |
| 8. Decision making |  |
| What decisions/recommendations will be open to the DMC | Possible recommendations could include:   - No action needed, trial continues as planned - Early stopping due, for example, to clear benefit or harm of a treatment, futility, or external evidence - Extending recruitment (based on actual control arm response rates being different to predicted rather than on emerging differences) or extending follow-up |
| The role of formal statistical methods, specifically which methods will be used and whether they will be used as guidelines or rules | There will be no interim analysis |
| How decisions or recommendations will be reached within the DMC | Recommendations will be based on agreement.  It is important that the implications (eg ethical, statisticial, practical, financial) for the trial be considered before any recommendation is made. |
| When the DMC is quorate for decision-making | Effort will be made for all members to attend or to respond by mail. |
| Can DMC members who cannot attend the meeting input | In case of a face to face meeting: the report is circulated before the meeting, DMC members who will not be able to attend the meeting may pass comments to the DMC Chair for consideration during the discussions. |
| What happens to members who do not attend meetings | If a member does not attend a meeting, it should be ensured that the member is available for the next meeting. If a member does not attend a second meeting, they should be asked if they wish to remain part of the DMC. If a member does not attend a third meeting, they should be replaced. |
| Whether different weight will be given to different endpoints (eg safety/efficacy) |  |
| Any specific issues relating to the trial design that might influence the proceedings, eg cluster trials, equivalence trials, multi-arm trials |  |
| 9. Reporting |  |
| To whom will the DMC report their recommendations/decisions, and in what form | The Trial Steering Committee. |
| Whether minutes of the meeting be made and, if so, by whom and where they will be kept | Not routinely, only if preferred by the DMC |
| What will be done if there is disagreement between the DMC and the body to which it reports | If the DMC has serious problems or concerns with the Trial Steering Committee decision a meeting of these groups will be held. The information to be shown would depend upon the action proposed and the DMC’s concerns. Depending on the reason for the disagreement confidential data will often have to be revealed to all those attending such a meeting. The meeting should be chaired by a senior member of the trials office staff or an external expert who is not directly involved with the trial. |
| 10. After the trial |  |
| Publication of results | At the end of the trial there may be a meeting to allow the DMC to discuss the final data and give advice about data interpretation |
| The information about the DMC that will be included in published trial reports | DMC members will be named and their affiliations listed in the main report, unless they explicitly request otherwise. |
| Whether the DMC will have the opportunity to approve publications, especially with respect to reporting of any DMC recommendation regarding termination of a trial | The DMC may wish to be given the opportunity to read and comment on any publications before submission. |
| Any constraints on DMC members divulging information about their deliberations after the trial has been published | The DMC may discuss issues from their involvement in the trial when permission is agreed with the overseeing committee. |

**Supplementary file 8**

**Example of informed consent form of the TREAT EARLIER trial**

- I have read the information letter for trial participants. I could ask additional questions. My questions are answered. I had enough time to decide whether I would participate.
- I decided to participate in this study.
- I know that participating is completely voluntary. I know that I can decide to stop with this study at any time and without any reason.
- I give permission to inform my general practitioner about my participation in this study.
- I give permission to inform my rheumatologist about my participation in this study.
- I am aware that some persons can see my personal information. Those individuals are listed in the general brochure. I give permission for this inspection by these persons.
- I give permission to use obtained my data for the purposes mentioned in the information letter.
- I give permission to use obtained MRI data for the purposes mentioned in the information letter.
- I give permission to use obtained blood to investigated risk factors that are related to the development of rheumatic disease, such as rheumatoid arthritis.
- I give permission to inform my general practitioner/rheumatologist in case unexpected findings are identified that are or could be important for my own healthcare.
- I give/do not give* permission to store and use my data for future rheumatological research.
- I give/do not give* permission to store my body material for future research as mentioned in the information letter.

Name participant: (fill out yourself in block letters)
Signature: Date : __ / __ / __

-----------------------------------------------------------------------------------------------------------------

**Part below to complete by the researcher or his/her representative.**

I hereby declare that I have fully informed the participant about this study.
If during the study new insights become available that could influence the consent of the participant I will timely notify him/her.

Name researcher (or his/her representative):
Signature: Date: __ / __ / __

-----------------------------------------------------------------------------------------------------------------

Additional information is given by (if applicable):

Name:
Function:
Signature: Date: __ / __ / __

-----------------------------------------------------------------------------------------------------------------

* Strikethrough what does not apply.

**References**

1. Ostergaard M, Peterfy C, Conaghan P, McQueen F, Bird P, Ejbjerg B, et al. OMERACT Rheumatoid Arthritis Magnetic Resonance Imaging Studies. Core set of MRI acquisitions, joint pathology definitions, and the OMERACT RA-MRI scoring system. J Rheumatol. 2003;30(6):1385-6.

2. Stomp W, Krabben A, van der Heijde D, Huizinga TW, Bloem JL, van der Helm-van Mil AH, et al. Aiming for a shorter rheumatoid arthritis MRI protocol: can contrast-enhanced MRI replace T2 for the detection of bone marrow oedema? European radiology. 2014;24(10):2614-22.

3. Schmid MR, Hodler J, Vienne P, Binkert CA, Zanetti M. Bone marrow abnormalities of foot and ankle: STIR versus T1-weighted contrast-enhanced fat-suppressed spin-echo MR imaging. Radiology. 2002;224(2):463-9.

4. Mayerhoefer ME, Breitenseher MJ, Kramer J, Aigner N, Norden C, Hofmann S. STIR vs. T1-weighted fat-suppressed gadolinium-enhanced MRI of bone marrow edema of the knee: computer-assisted quantitative comparison and influence of injected contrast media volume and acquisition parameters. Journal of magnetic resonance imaging : JMRI. 2005;22(6):788-93.

5. Sudol-Szopinska I, Jurik AG, Eshed I, Lennart J, Grainger A, Ostergaard M, et al. Recommendations of the ESSR Arthritis Subcommittee for the Use of Magnetic Resonance Imaging in Musculoskeletal Rheumatic Diseases. Seminars in musculoskeletal radiology. 2015;19(4):396-411.

6. Bloem JL, Reijnierse M, Huizinga TWJ, van der Helm-van Mil AHM. MR signal intensity: staying on the bright side in MR image interpretation. RMD open. 2018;4(1):e000728.

7. Boer AC, Burgers LE, Mangnus L, Ten Brinck RM, Nieuwenhuis WP, van Steenbergen HW, et al. Using a reference when defining an abnormal MRI reduces false-positive MRI results-a longitudinal study in two cohorts at risk for rheumatoid arthritis. Rheumatology (Oxford, England). 2017;56(10):1700-6.

8. van Steenbergen HW, Mangnus L, Reijnierse M, Huizinga TW, van der Helm-van Mil AH. Clinical factors, anticitrullinated peptide antibodies and MRI-detected subclinical inflammation in relation to progression from clinically suspect arthralgia to arthritis. Ann Rheum Dis. 2016;75(10):1824-30.

9. Mangnus L, van Steenbergen HW, Reijnierse M, van der Helm-van Mil AH. Magnetic Resonance Imaging-Detected Features of Inflammation and Erosions in Symptom-Free Persons From the General Population. Arthritis Rheumatol. 2016;68(11):2593-602.
